# Leadership Lessons From a Quality Improvement Project to Decrease the Avoidable Days in the Hospital Related to Decision-Making Capacity

**DOI:** 10.7759/cureus.99315

**Published:** 2025-12-15

**Authors:** Hira Fatima, Syed Atif, Bella Ayala, Zorez R Mian, Sobia Zareen, Erum Azhar, Abdul Waheed

**Affiliations:** 1 Family Medicine, WellSpan Good Samaritan Hospital, Lebanon, USA; 2 Family and Community Medicine, Creighton University School of Medicine, Phoenix, USA; 3 Medicine, CMH Lahore Medical College and Institute of Dentistry, Lahore, PAK; 4 Family and Community Medicine, Baylor College of Medicine, Houston, USA; 5 Obstetrics and Gynecology, Dignity Health East Valley, Gilbert, USA; 6 Obstetrics and Gynecology, Creighton University School of Medicine, Phoenix, USA; 7 Family Medicine, Dignity Health Medical Group, Gilbert, USA

**Keywords:** avoidable days in the hospital, decision-making capacity, hospital length of stay, leadership, u-care assessment tool

## Abstract

Introduction: Extended hospital stays drive up costs for patients, payors, and health systems. For this reason, it is crucial to minimize “avoidable days” (ADs), or delays to discharge after a patient is medically stable. Delays in determining decision-making capacity are a significant contributor to ADs. The present study implemented the U-CARE tool - a structured, evidence-informed capacity assessment instrument that provides a standardized framework for evaluating understanding, choice, appreciation, and reasoning. The tool requires brief clinician training and has been used in prior hospital-based quality improvement initiatives to improve consistency in capacity evaluations. We introduced U-CARE as part of a multifaceted bundled intervention to decrease ADs at a community hospital in Lebanon, Pennsylvania.

Methods: A quasi-experimental pre-post intervention study was conducted, involving a review of trends in ADs in pre- and post-intervention periods. ADs in the pre-intervention period were compared with ADs in the post-intervention period. There were three components of the multifaceted intervention. First, the quality improvement team including the co-authors of this article educated all attending physicians and residents on the use of the U-CARE assessment tool. Second, case managers performed weekly audits of patient charts and sent reminders about ADs to the hospitalist physician. Third, multidisciplinary rounds (MDR) included a scripted inquiry about ADs to prompt physicians to use the U-CARE tool.

Results: The pre-intervention survey had a response rate of 100% (n=32). Of note, 87.5% of respondents indicated that they are challenged with decision-making capacity evaluation on a patient. 61.5% were not utilizing any capacity assessment tool and 42.3% were not aware of the U-CARE tool for capacity evaluation. In response to inquiry about common reasons for ADs in patients needing capacity assessment, 48.2% indicated delays in psych consults, whereas 44.8% attributed it to not having a standardized process in place. The data on ADs was analyzed using the Statistical Process Control (SPC) Chart with the software JMP Pro 16 (JMP Statistical Discovery LLC, Cary, NC, USA). One-way ANOVA was performed to detect statistically significant (p value<0.05) differences in ADs between the three phases. Pre-intervention phase showed an average of 220 ADs per month.

Conclusions: The phase analysis on the SPC chart shows that leadership change had a significant association with the outcomes. Further research is needed to assess the efficacy of U-CARE tool implementation in reducing ADs and to elucidate the impact of leadership presence on successful implementation.

## Introduction

Hospital length of stay (LOS) is a crucial indicator of system performance, directly linked to costs, bed availability, and patient outcomes. Prolonged LOS is associated with higher risks of hospital-acquired complications, functional decline, and readmissions, while also congesting flow from the emergency department (ED) and limiting access for incoming patients. Common drivers include patient factors (age, comorbidity, functional and cognitive status), care-process issues (diagnostic/treatment delays, complications), and system barriers (discharge-planning gaps, post-acute placement delays, and care-coordination failures). Large reviews consistently identify discharge bottlenecks, especially arranging post-acute services and social supports, as major contributors to extended stays [1-3].

Within this broader context, Majeed et al. [1] define avoidable days (ADs) as inpatient days that occur after a patient is medically stable but remains hospitalized due to non-medical delays. ADs commonly stem from placement or insurance authorization delays, transportation issues, and workflow/communication gaps. Importantly, decision-making capacity (DMC) uncertainty can also stall discharge: when a patient’s ability to understand, appreciate, reason about, and express a choice is unclear, teams may defer to psychiatry or legal processes, pathways that are not scalable for every case and can add days to LOS [4].

The ethical stakes are high; clinicians must balance respect for autonomy with the duty to protect vulnerable patients. Appelbaum outlines DMC across four abilities: communicating a choice, understanding relevant information, appreciating the consequences, and reasoning about options [5]. It cautions against relying on global cognitive screens like the Mini-Mental State Examination (MMSE), as no single cutoff provides adequate sensitivity and specificity for capacity determinations. Consistent with this, Grisso and Appelbaum [6] advocate a structured bedside approach grounded in these abilities rather than cognition scores alone; empirical work also shows that MMSE is an imprecise discriminator for DMC [7].

Published outcome studies isolating Understanding, Consistency, Appreciation, Reasoning, Expression (U-CARE) specifically are limited; peer-reviewed work more commonly evaluates closely related Understanding, Appreciation, Reasoning, Expression (U-ARE) protocols derived from the same four-abilities framework. In memory-care and research settings, U-ARE has been associated with more efficient, standardized capacity assessments and with “capacity optimization” strategies (e.g., communication aids) that preserve autonomy [8]. In long-term care and community practice, descriptive quality improvement (QI) reports using U-CARE-style checklists echo these benefits, clearer bedside documentation and faster team consensus even though controlled trials remain scarce [4,5].

Finally, because implementation succeeds or fails at the team level, we incorporated multidisciplinary rounds (MDR) prompts and shared accountability. Evidence from a Cochrane review by Silva and colleagues indicates that collective leadership approaches can improve professional practice and healthcare outcomes, supporting the leadership and teamwork elements embedded in our intervention [9].

The present study implemented a pragmatic capacity-assessment approach using the U-CARE/U-ARE framework (a structured bedside method aligned with the four abilities) and bundled system supports (education, auditing/reminders, and MDR prompts). The objective of this project was to evaluate whether a structured U-CARE-based workflow could reduce ADs related to these delays at a community hospital in Lebanon, Pennsylvania.

## Materials and methods

Study design and setting

A quasi-experimental pre-post quality‐improvement study was conducted at a community hospital in Lebanon, Pennsylvania. Monthly ADs were tracked across three phases: pre-intervention (August 2021-June 2022), roll-out (June-September 2022), and post-intervention (September 2022-June 2023). The project was reviewed by the institution and determined to be a quality improvement, exempt from IRB oversight.

Study population

The cohort included all adult inpatients on hospitalist services for whom ADs are routinely recorded by case management using the hospital’s operational definition (days spent in hospital after medical stability until discharge). In addition to overall ADs, a subset of ADs attributed to capacity-related delays was identified from case-management annotations and MDR notes.

Pre-intervention

**Figure 1:**
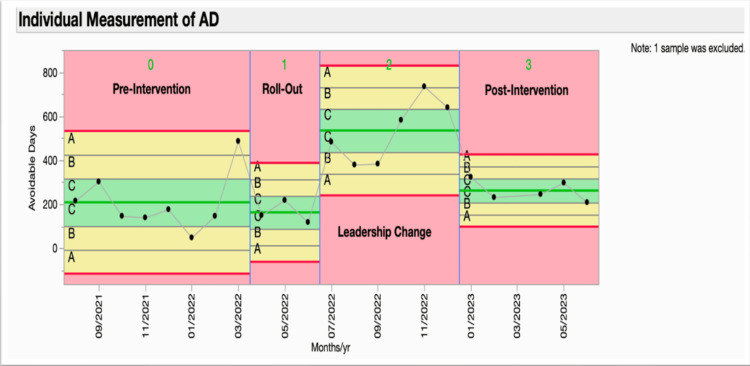
Trends in avoidable days (ADs) Statistical process control (SPC) phase analysis control chart tracking the scholarly activities completed by individual residents, divided into Pre-intervention (0) and Post-intervention (1) phases separated by the (blue) intervention line. μ0 represents the mean as the center green line. UCL (upper red line): three sigma upper control limit. LCL (lower red line): three-sigma lower control limit. Values in UCL and LCL represent three standard deviations above and below the mean, respectively. Special cause variation is highlighted in red with the Nelson rule number 1 and a change in the mean from the pre-intervention to the post-intervention phase. UCL: upper control limit, LCL: lower control limit

**Figure 2:**
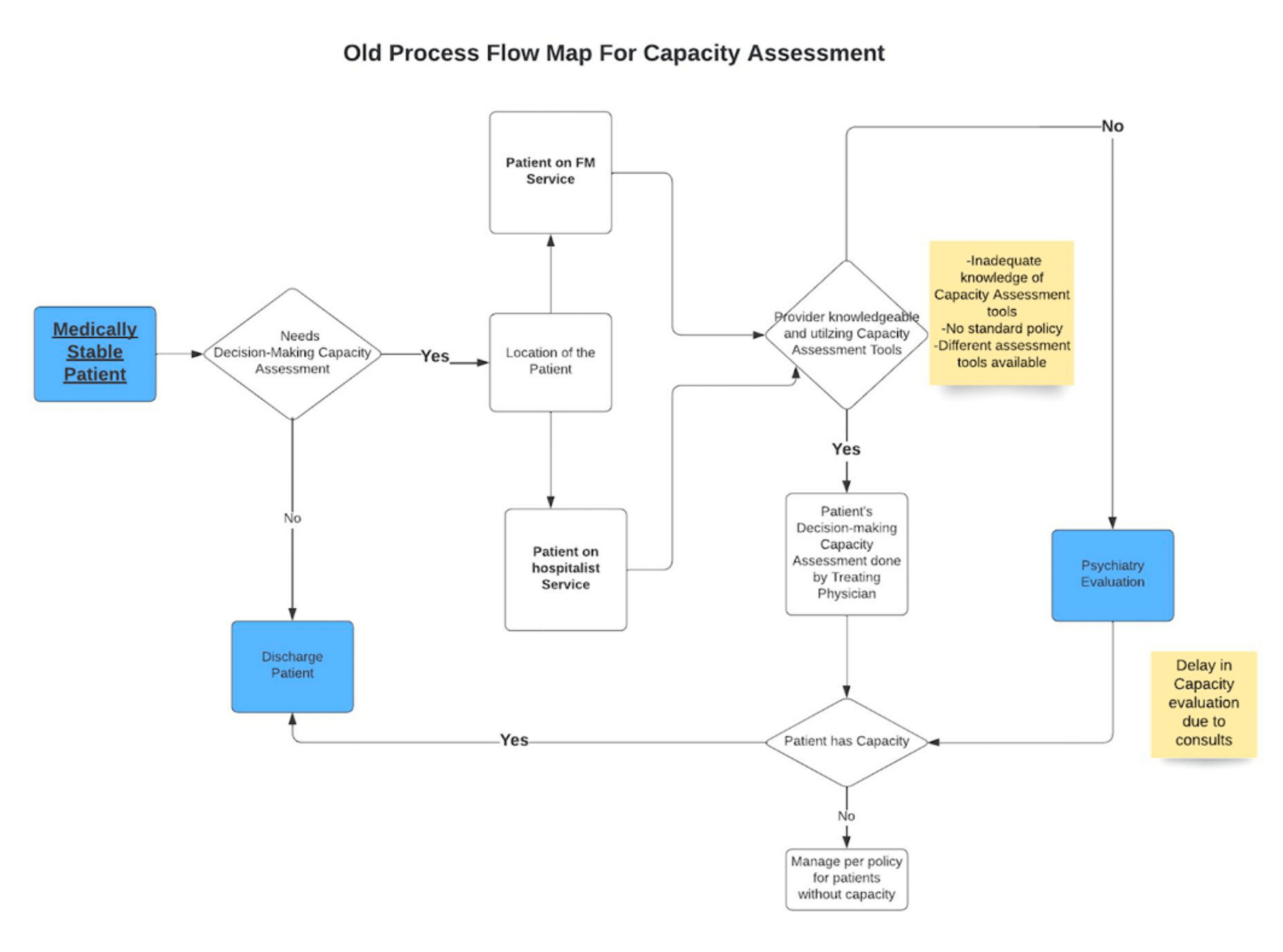
Baseline Process Map FM = Family Medicine

A pre-intervention baseline of monthly ADs (see Figure 1) and a pre-intervention process map of the existing DMC workflow (see Figure 2) were developed to identify failure points and handoffs.

**Figure 3:**
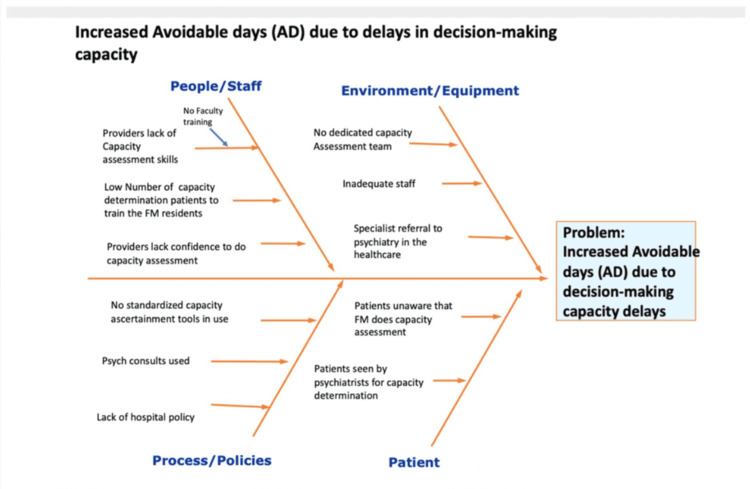
Root Cause Analysis FM = Family Medicine

A root cause analysis (RCA) was conducted using an Ishikawa (fishbone) diagram with representation from hospital medicine, family medicine residency, case management, nursing leadership, and social work (see Figure 3). Contributing factors were organized under People, Policies, Procedures, Environment, and Communication/Information flow. Outputs from the RCA informed intervention selection and the Knowledge, Attitudes, and Practices (KAP) survey content.

A cross-sectional KAP survey was administered to attending physicians and residents on inpatient services, achieving a 100% response rate (n=32). Survey domains included 1) Knowledge: awareness of capacity frameworks/tools (e.g., U-CARE), familiarity with Appelbaum’s four abilities; 2) Attitudes: perceived difficulty of DMC evaluations; beliefs about the role of psychiatry/courts; perceived ethical risks (autonomy vs protection); and 3) Practices/Barriers: current use of any standardized tool; turnaround times for psychiatry consults; presence/clarity of local policies; documentation practices.

**Figure 4:**
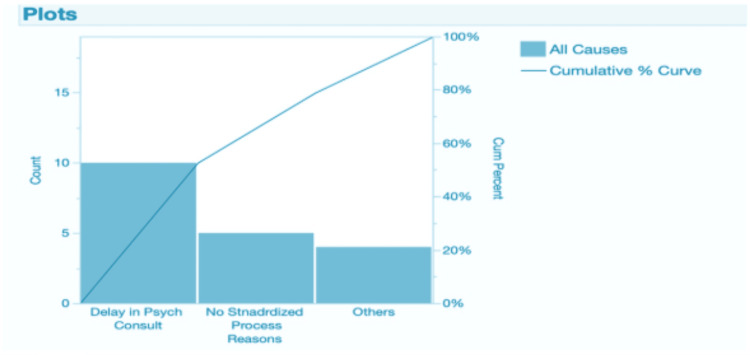
Pareto Chart from the Baseline Knowledge, Attitudes, and Practices (KAP) Survey

Results were summarized descriptively (proportions) and visualized with a Pareto chart to identify the “vital few” drivers (see Figure 4). Key pre-intervention findings informing the intervention included: high self-reported challenge with DMC evaluation (87.5%), low use of any capacity tool (61.5% not using one), limited awareness of U-CARE (42.3% unaware), and dominant barriers of psych consult delays (≈48%) and lack of a standardized process (≈45%).

Intervention

The present study introduced a multifaceted intervention centred on a structured bedside framework labelled U-CARE (Understanding, Consistency, Appreciation, Reasoning, Expressing a choice).

Education

The Director of Hospital Medicine provided education sessions for all attendings and residents on the U-CARE approach and expectations for bedside assessment and documentation.

Audit and Feedback

Case managers conducted weekly chart audits of open cases with ADs and sent targeted reminders to the hospitalist of record regarding capacity-related delays.

Workflow Prompt

MDR adopted a scripted inquiry about ADs and DMC status to prompt timely application of U-CARE and escalation only when needed.

Process fidelity was monitored by logging delivery of education sessions, completion of weekly audits, and MDR prompt uptake.

Data collection and analysis

Data were collected on outcome measures (ADs during various phases) and process measures (a three-part intervention). Statistical Process Control (SPC) was used to display and monitor ADs over time. Monthly ADs were plotted using an Individuals (XmR) chart, a type of SPC chart used to track individual data points over time, in JMP® Pro 19 (JMP Statistical Discovery LLC, Cary, NC, USA). Institute for Healthcare Improvement (IHI) special-cause rules were applied to detect non-random variation (e.g., shifts, trends) and to recalculate centerlines/control limits when indicated. For phase-level comparisons, we conducted a one-way ANOVA of monthly AD means across the three phases (α = 0.05). Analyses focused on both overall ADs and capacity-attributable ADs where available.

## Results

**Figure 5:**
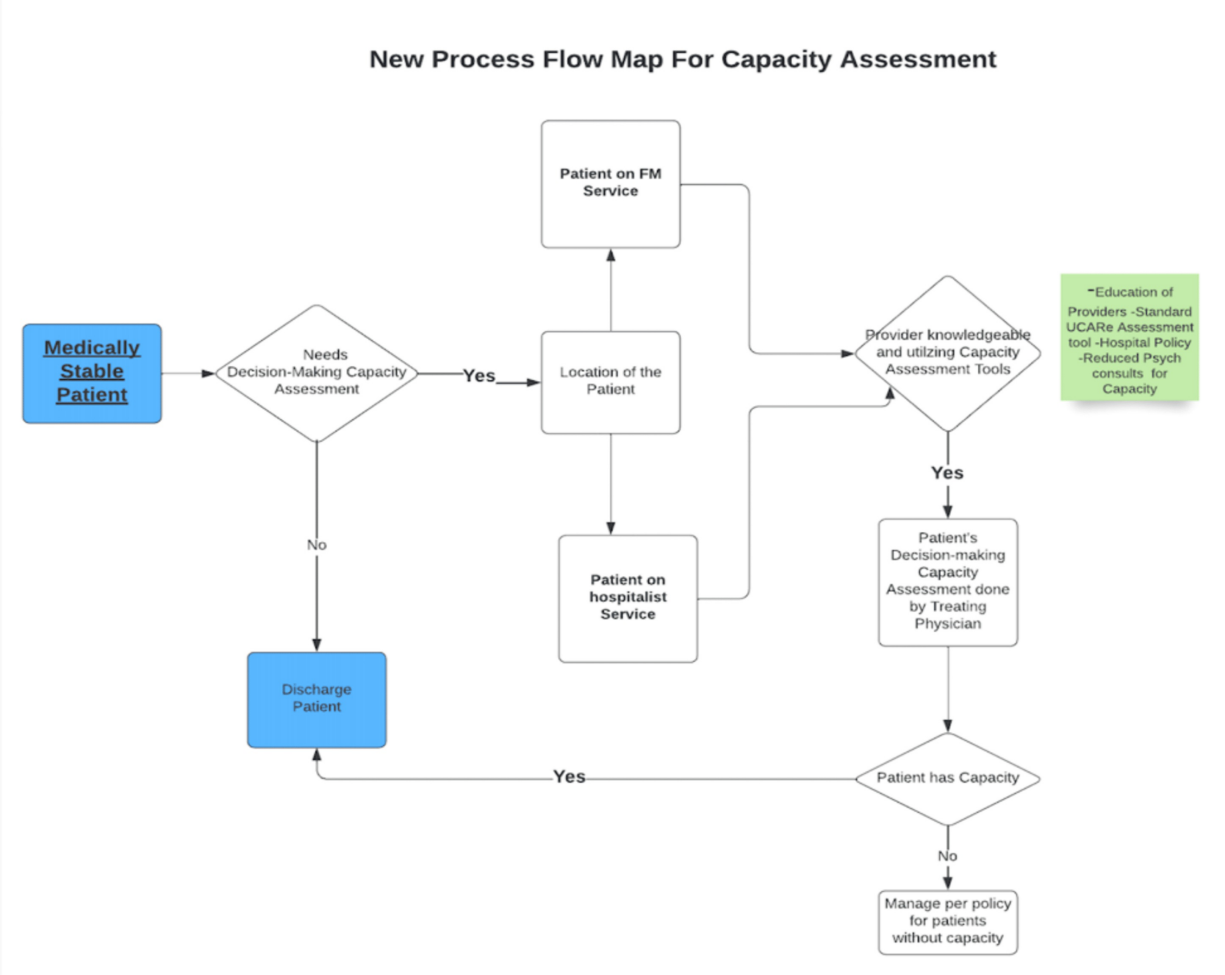
Post-implementation Process Map FM = Family Medicine; MDR = Multidisciplinary Rounds; U-CARE = Understanding, Consistency, Appreciation, Reasoning, Expressing a Choice.

The post-intervention process map (Figure 5) illustrates the redesigned bedside pathway for DMC. It includes an early U-CARE prompt at the point of clinical stability, clear documentation expectations, and a scripted MDR check to identify capacity-related barriers before ADs accrue. Compared with the baseline map (Figure 2), redundant handoffs were removed and escalation criteria clarified. 

The pre-intervention baseline period demonstrated that mean ADs were 208 per month (Figure 1). This value functioned as the reference centerline on the statistical process control (SPC) chart and provided a benchmark against which subsequent phase changes were evaluated.

The initial implementation period showed an immediate beneficial effect of the bundled intervention. During Phase 1, mean ADs decreased to 163 per month, representing a reduction of 45 days and a 21.6% improvement compared with baseline. In the Individuals (XmR) SPC chart, Phase-1 values consistently fell below the baseline centerline and met Institute for Healthcare Improvement special-cause criteria, prompting recalculation of the centerline. This reduction corresponded with the period in which education sessions, weekly case-management audits, and MDR prompts were most consistently executed, aligning with the optimized workflow illustrated in Figure 5.

The subsequent leadership transition period was associated with a marked deterioration in performance. During Phase 2, mean ADs increased to 534 per month the highest level observed across all phases. This increase represented a rise of 371 ADs compared with Phase 1 (+227.6%) and 326 ADs compared with baseline (+156.7%). The SPC chart displayed a clear upward level shift with rule-based evidence of special cause variation, indicating that the change in hospital medicine leadership corresponded with a substantial disruption in the implementation of standardized processes.

The period following leadership re-engagement demonstrated partial stabilization and improvement. During Phase 3, mean ADs decreased to 262 per month, which was 272 lower than the Phase-2 peak (-50.9%). Although performance improved, ADs remained 54 above baseline (+26.0%) and 99 above the Phase-1 mean (+60.7%). The XmR chart reflected a downward shift consistent with restoration of partial process control, though not a full return to the initial gains achieved during early implementation.

The survey-observed mechanisms provided insight into the drivers underlying these trends. Before implementation, the KAP survey (Figure 4) showed that most clinicians found capacity assessment challenging (87.5%), did not use a formal assessment tool (61.5%), and were unaware of U-CARE (42.3%). The Pareto chart identified delays in psychiatry consultation (48%) and the absence of a standardized workflow (45%) as the most influential contributors to capacity-related ADs. Additionally, the fishbone diagram (Figure 3) highlighted gaps in policy clarity, documentation variability, and communication flow all issues directly addressed by the intervention components.

The comparison of mean ADs across phases demonstrated a statistically significant difference. A one-way ANOVA yielded an F value of 11.4 (p < 0.05) (Table 1), confirming that the observed variation across phases was unlikely due to chance. The direction and magnitude of changes initial reduction, subsequent deterioration with leadership turnover, and partial recovery with leadership engagement support the interpretation that the intervention was effective when supported by stable leadership, but vulnerable to disruption when leadership continuity was interrupted.

**Table 1:** One-Way ANOVA Results Showing the Effect of Intervention on the Outcome Variable Std Error uses a pooled estimate of error variance. 1 row excluded from analysis.

Analysis of Variance					
Source	DF	Sum of Squares	Mean Square	F Ratio	Prob > F
Intervention	3	460,478.29	153,493	11.4010	0.0002*
Error	18	242,336.30	13,463		
C. Total	21	702,814.59			
Means for Oneway ANOVA					
Level	Number	Mean	Std Error	Lower 95%	Upper 95%
0	8	208.250	41.023	122.06	294.44
1	3	162.667	66.990	21.93	303.41
2	6	534.333	47.369	434.81	633.85
3	5	261.800	51.891	152.78	370.82

## Discussion

Our intervention demonstrated that a bundled strategy incorporating the U-CARE decision-making capacity workflow, audit and feedback, and MDR team-based inpatient care meetings was associated with an initial reduction in ADs. Following a transition in hospital medicine leadership, ADs increased beyond baseline, then declined again after the new leader re-engaged with the work. This pattern highlights the central role of consistent leadership involvement in sustaining quality improvement efforts and maintaining reductions in ADs. Suggestive of two complementary insights: (1) structured capacity tools can reduce avoidable delays, and (2) leadership continuity and institutional embedding are critical to sustain those gains.

Majeed et al. [1] showed that delays, often unrelated to ongoing medical care, inflate bed occupancy and prolong stays. Broader work by Allen et al. [10]. highlighted delays to placement barriers, fragmented coordination, and process inefficiencies. In a systematic review Cadel et al. [2] reported that discharge facilitation and patient-tracking systems can help in reducing delays, but their effectiveness was contingent on organizational commitment and leadership support. Broader analyses by Abdelhalim et al. [11] concluded that delayed discharge persists because of structural barriers, leadership gaps, and weak cross-setting integration. Studies indicate that nearly 6% of internal medicine inpatients experienced delayed discharge, with contributions from dependence in ADLs and hospital-acquired complications [12]. Delayed discharge has been closely linked to overall hospital length of stay and call for hospital process improvements paired with post-acute capacity [13].

Decision-making capacity represents a specific discharge clinical barrier. Appelbaum’s “four abilities” model, understanding, appreciation, reasoning, and expressing a choice remains the dominant clinical framework [5]. Expansions on this model have linked these domains to their legal and ethical roots, highlighting the importance of structured assessment in clinical practice [14]. Global cognitive screens are insufficient for capacity: Kim and Caine showed the MMSE lacks sensitivity for assessing appreciation and reasoning, leading to misclassification [7]. To operationalize the abilities at the bedside, U-ARE offers a pragmatic, stepped approach; Hamilton et al. reported more efficient, standardized assessments and “capacity optimization” tactics that preserve autonomy [8]. Our Phase-1 AD reduction is consistent with these data: embedding a structured, ability-based method (here, U-CARE-aligned) reduced uncertainty, standardized documentation, and surfaced barriers earlier in MDR.

The Phase-2 spike coincided with leadership turnover, then improved with leader re-engagement, mirroring evidence that leadership behavior shapes patient safety and quality, and that performance erodes when sponsorship lapses [14,15]. Collective leadership approaches further show gains in professional practice, outcomes, and staff well-being [16]. In Kotter’s terms, Phase-1 reflected urgency, a guiding coalition (director, case management, MDR), and short-cycle wins; turnover disrupted that coalition, and Phase-3 recovery followed re-forming it. Healthcare applications of Kotter’s 8-step model similarly associate explicit attention to coalition-building, barrier removal, and anchoring in culture with improved adoption and sustainment [17-19].

Finally, beyond DMC workflows, hospital medicine leadership influences LOS. Program evaluations and reviews link hospitalist models, paired with reliable discharge planning and daily multidisciplinary goal setting, to shorter LOS and better satisfaction [20-23]. Our bundle operationalized those principles (MDR scripts, closed-loop audits, standard work), and loss then restoration of visible leadership tracked closely with AD performance.

For organizations adopting U-CARE/U-ARE-style capacity pathways, pair the technical change with a leadership-proof sustainment plan: (i) anchor a Kotter-style guiding coalition beyond a single leader; (ii) hard-wire MDR prompts and documentation standards; (iii) maintain a shared LOS/AD dashboard owned by hospital medicine and case management; and (iv) monitor capacity-attributable ADs to verify mechanism and target coaching. Standardization reduces variation; stable, engaged leadership keeps the gains.

This quality improvement project has several limitations. First, the observed association between leadership presence and reductions in ADs may be influenced by unmeasured confounders, such as concurrent workflow changes, staffing patterns, or seasonal variation in patient volume. Second, the project was conducted at a single community hospital, which may limit generalizability to other settings with different resources, structures, or decision-making processes. Finally, the pre-post design does not allow for causal inference. Despite these limitations, the findings suggest that formal leadership involvement plays an important role in sustaining improvements in discharge processes and reducing ADs.

## Conclusions

Formal leadership presence is significantly associated with outcomes in this quality improvement project. The U-CARE tool has the potential to reduce ADs by allowing hospitalists to determine decision-making capacity without a psychiatry consult. The benefits of eliminating ADs would be experienced by healthcare systems, payors, and patients alike.
